# The effect of motion on IMRT – looking at interplay with 3D measurements

**DOI:** 10.1088/1742-6596/444/1/012049

**Published:** 2013

**Authors:** A Thomas, H Yan, M Oldham, T Juang, J Adamovics, FF Yin

**Affiliations:** 1 Duke University Medical Center, Durham, NC, USA; 2 Rider University, Lawerenceville, NJ, USA

## Abstract

Six base of skull IMRT treatment plans were delivered to 3D dosimeters within the RPC Head and Neck Phantom for QA verification. Isotropic 2mm 3D data was obtained using the DLOS-PRESAGE system and compared to an Eclipse (Varian) treatment plan. Normalized Dose Distribution pass rates were obtained for a number of criteria. High quality 3D dosimetry data was observed from the DLOS system, illustrated here through colormaps, isodose lines, profiles, and NDD 3D maps. Excellent agreement with the planned dose distributions was also observed with NDD analysis revealing > 90% NDD pass rates [3%, 2mm], noise < 0.5%. This paper focuses on a detailed exploration of the quality and use of 3D dosimetry data obtained with the DLOS-PRESAGE system.

## 1. Introduction

The effect of interplay has been well documented and written into TG reports with warnings of its negative consequences[1–6]. However, the effect has only been observed in 2D or a series of 2D devices, and has never been measured with a truly 3D device. Research on a 3D device may lead to different conclusions as to the outcome and possible consequences of motion management with leaf motion present. This work shows our initial investigations of interplay as documented by a 3D dosimeter building on the work of Brady *et al* [7].

## 2. Methods

Isotropic 2mm 3D dosimetry data was measured using the Presage/DLOS (Duke Large Field-of-view Optical-CT Scanner) [8]. Measured data was compared to eclipse calculation (AAA algorithm) in the CERR software[9]. Comparison methods included comparing isodose lines and dose-volume-histograms (DVH).

### 2.1. Dosimeter/Phantom

**Figure 1:**
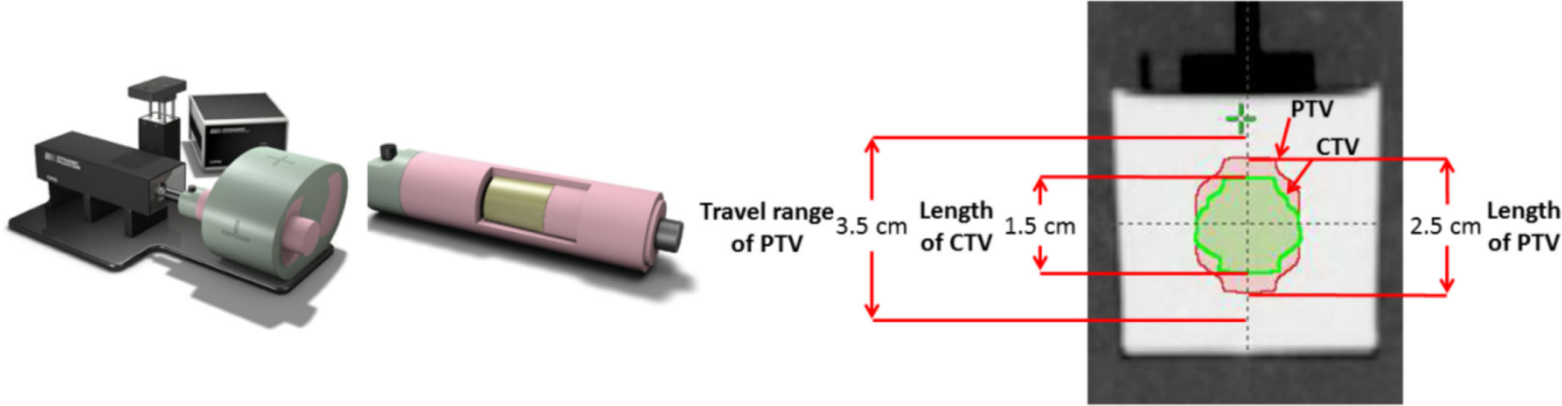
CIRS motion phantom (left) and dosimeter insert (middle) with CT of dosimeter and GTV/CTV and ITV/PTV volumes contoured (right).

A 5cm diameter, 5cm long PRESAGE cylinder was placed within a CIRS dynamic thorax phantom[7], see Fig 1. The phantom was set to oscillate with a period of 4s and an amplitude of ±5mm. The CIRS phantom-dosimeter combination was scanned on a GE Lightspeed X-ray-CT scanner with a 1.25mm slice thickness. Contours were made on the CT for a GTV/CTV and then expanded in the direction of motion creating an ITV/PTV as shown in Fig 1. From these contours a 5-field, 12 Gy IMRT and a 1-arc, 12 Gy RapidArc treatment plans were created for the phantom-dosimeter combination. Calculation was performed with the Eclipse planning system, using the Analytic-Anisotropy-Algorithm (AAA), with 2.5mm^3^ isotropic resolution, with the heterogeneity correction applied.

The delivery of the plans occurred on a Novalis Tx. The delivery of each plan (IMRT & RapidArc) occurred twice; once with the motion phantom in a static position acting as a control, and once with the phantom in motion using the parameters stated above.

### 2.2. 3D Data Acquisition

3D dose measurements were performed with the cylindrical PRESAGE^®^ dosimeters which were compatible with the CIRS dynamic phantom. PRESAGE^®^ (Heuris Pharma, Skillman, NJ) is a polyurethane based dosimetry material which changes color when exposed to ionizing radiation, causing an optical density change (ΔOD) for visible light. The radiation induced ΔOD is proportional to the locally absorbed dose, and can be imaged using optical-CT[10–12]. Recent comprehensive evaluations have shown PRESAGE^®^ to be an excellent material for accurate 3D dosimetry [13, 14].

To obtain the delivered 3D dose distribution, the radiation-induced ΔOD throughout the dosimeter was imaged using the DLOS system). The ΔOD is determined by reconstructing corrected projection images utilizing parallel beam filtered back-projection as described in [8]. 3D data are acquired through the accumulation of 360 projection images over 360° to meet Nyquist sampling criteria for 2mm^3^ isotropic reconstructions. Each projection image is captured 20 times and averaged to increase the SNR at each angle. The projection images are dark noise and flood field corrected to reduce the effects of particulates on glass surfaces, non-uniformity of CCD pixel response, dark current and readout noise.

### 2.3. 3D IMRT QA Data Analysis

The 3D ΔOD distribution was converted to dose by normalizing the IMRT static delivery to the treatment plan and using that normalization value to scale the remaining dosimeters. Upon normalization of the data DVHs and isodose overlays of the plan, static and motion deliveries were created to quantify the interplay effect in 3D.

**Figure 2:**
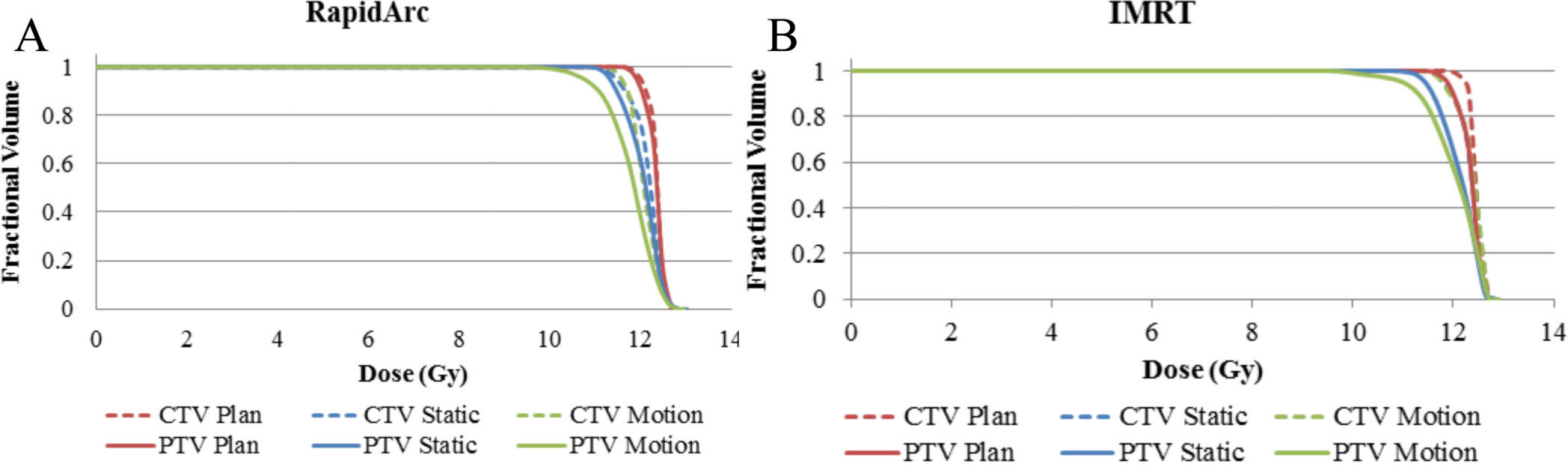
DVHs of (A) The RapidArc plan and (B) The IMRT plan. In both instances dashed lines indicate GTV/CTV coverage and solid lines represent ITV/PTV coverage.

Figure 2 shows results of the DVH plots according to the contours used in creating the treatment plans. Inspection of the figure shows the least coverage for the ITV/PTV volumes with the motion delivery. Of interest, however, the GTV/CTV coverage is on par with the stationary delivery meaning the expanded volumes performed their purpose even with interplay. This was expected to break down due to interplay, but given the relatively low modulation of the beams and small volume irradiated the effect was not as dramatic as seen previously by other authors[1, 3, 6].

## 3. Results

### 3.1. DVH Analysis

### 3.2. Isodose Map

**Figure 3:**
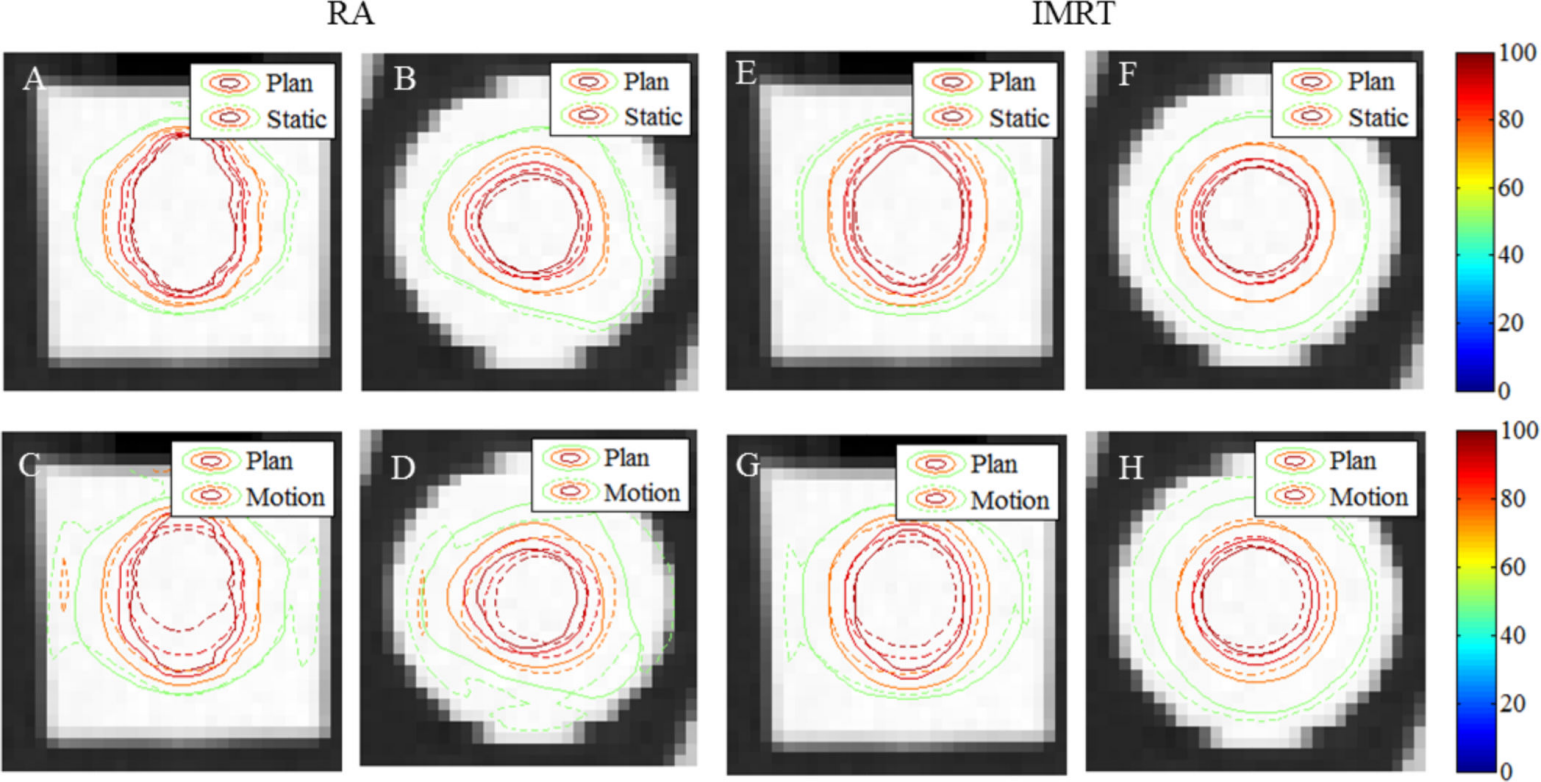
Static (Top Row) and Motion (Bottom Row) delivery overlaying the planned Rapid Arc and IMRT deliveries. The 1^st^ column is the coronal view and the 2^nd^ is the axial view. The colorbar indicates dose delivered in % of maximum planned dose. In all plots above the solid lines equal the planned doses and the dashed lines equal the measured doses.

Figure 3 shows the RapidArc and IMRT axial and coronal isodose overlays of the planned delivery with the static and motion deliveries.

Inspection of figure 3 shows the results of interplay in the coronal and axial planes. In both the RapidArc and IMRT cases, the Static delivery (dashed lines – top row) matches the Planned delivery (solid lines – bottom row) to within a few millimeters, however the motion cases, significant deviation in the form of shifting and stretching are seen – leading to under-dosing of the PTV as seen in the DVHs. The plans with motion also appear to be noisier, but it is not due to the readout. Given the motion during delivery this is simply a redistribution of dose creating a more chaotic distribution. The noise in all dosimeters was < 0.5% of the high dose regions.

## 4. Conclusions

The data acquired for this work was both promising and initially surprising. 3D data documenting interplay with motion during delivery has been acquired with high quality and 2mm isotropic resolution. It was surprising in the sense that when expanding the GTV/CTV for motion, the drop in coverage of the volume was not significantly reduced as seen by a stationary delivery of the same volume. Perhaps this was simply due to the small volume. This work shows that further investigation into the effects of interplay can be done in 3D and perhaps allow for better understanding of motion effects with different frequencies (ie. Leaf speed, Breathing period, Gantry rotation, etc) and lead to updated recommendations on techniques of motion management.
